# Inflammation in preschool cystic fibrosis is of mixed phenotype, extends beyond the lung and is differentially modified by CFTR modulators

**DOI:** 10.1136/thorax-2024-221634

**Published:** 2025-02-10

**Authors:** Shivanthan Shanthikumar, Liam Gubbels, Anson Tsz Chun Wong, Hannah Walker, Sarath C Ranganathan, Melanie R Neeland

**Affiliations:** 1 Infection, Immunity and Global Health Theme, Murdoch Children's Research Institute, Parkville, VIC, Australia; 2 Department of Paediatrics, University of Melbourne, Parkville, VIC, Australia; 3 Respiratory and Sleep Medicine, Royal Children's Hospital, Parkville, VIC, Australia; 4 Children's Cancer Centre, Royal Children's Hospital, Parkville, VIC, Australia

**Keywords:** Cystic Fibrosis, Allergic lung disease, Innate Immunity, Cytokine Biology, Paediatric Lung Disaese

## Abstract

**ABSTRACT:**

**Background:**

Early-life inflammation has long been recognised as a key pathophysiological process in the evolution of cystic fibrosis (CF) lung disease. Despite this, no CF-specific anti-inflammatory treatments have been developed. This is crucial even in the era of highly effective modulator therapy as recent evidence suggests that modulators alter, but may not fully resolve, pulmonary inflammation.

**Methods:**

In this study, we used clinical microbiology data, high-dimensional flow cytometry and multiplex immunoassays to compare pulmonary (bronchoalveolar lavage (BAL)) and systemic immunity in 70 preschool children with CF and a total of 32 age-matched preschool controls.

**Results:**

We show that inflammation in the early-life CF lung is characterised by innate cell infiltration (neutrophils: 31.31 vs 1.8% of BAL in CF compared with controls, FDRp=0.0001; eosinophils: 0.55 vs 0.06%, FDRp=0.001, and monocytes: 1.91 vs 0.45%, FDRp=0.004) and widespread upregulation of both traditional and type 2 inflammatory soluble signatures (40 analytes significantly elevated in BAL of CF compared with controls, all FDRp<0.1). Key targetable features of this response included pulmonary interleukin (IL)-8 and IL-13 which were most significantly associated with neutrophilic and eosinophilic infiltration, respectively (IL-8 and neutrophils; Spearman rho=0.68, FDRp=0.002: IL-13 and eosinophils; Spearman rho=0.75, FDRp=0.01). Signatures of type 2 inflammation, as identified by REACTOME pathway analysis, including IL-4, IL-13 and FGF-2, were highly elevated in both the lungs and circulation in early CF. When exploring the efficacy of Cystic Fibrosis Transmembrane Conductance Regulator modulators to resolve pulmonary and systemic inflammation in early life, we showed that different classes of modulators have varying effects on inflammation, with ivacaftor showing a more significant effect in the lungs and circulation than lumacaftor/ivacaftor. Finally, we showed that CF children with pathogen colonisation had similar levels of pulmonary inflammation as CF children without pathogen colonisation (no significant differences), and that inflammation was evident during infancy even without evidence of colonisation (as observed by significant increases in levels of SDF-1alpha, M-CSF, IL-2, IL-9, IL-12p40, IL-17, MCP-1 and LIGHT/TNFSF14, all FDRp<0.1), highlighting a role for intrinsic dysregulation of inflammation that begins in early life.

**Conclusions:**

We provide a rationale for targeted anti-inflammatory intervention in early-life CF.

**WHAT IS ALREADY KNOWN ON THIS TOPIC**

- Inflammation is a fundamental component of cystic fibrosis (CF) lung disease, yet no anti-inflammatory treatments have been developed. Understanding the early-life origins and characteristics of inflammation is essential for the development of new therapies to ensure best long-term outcomes for people with CF.

**WHAT THIS STUDY ADDS**

- Inflammatory cell infiltration and widespread increases in type 2 soluble signatures are found in both the lungs and circulation of preschool children with CF. These responses arise independent of respiratory pathogen colonisation in the first two years of life and are partially restored by the Cystic Fibrosis Transmembrane Conductance Regulator modulator ivacaftor.

**HOW THIS STUDY MIGHT AFFECT RESEARCH, PRACTICE OR POLICY**

- We provide evidence for translating anti-inflammatory therapy into paediatric CF clinical care and highlight that strategies to monitor and protect cardiovascular health should be considered for children with CF.

## Introduction

 While the outlook for people with cystic fibrosis (CF) has dramatically improved in recent years, it remains a life-shortening condition where the primary source of morbidity and mortality is lung disease.^1^ It is well established that irreversible structural lung damage begins in early life for people with CF, and that the preschool years represent a crucial window for intervention to prevent the development of lung disease and ensure best long-term outcomes.^2 3^ Along with infection and impaired mucociliary clearance, inflammation has long been recognised as a key pathophysiological process in the evolution of CF lung disease.^1^ However, while CF-specific treatments targeting infection and mucociliary clearance are widely used, no such anti-inflammatory treatments have been developed.^4^

To develop anti-inflammatory therapies for young children with CF, it is essential to better understand the evolution of pulmonary inflammation in the infant and preschool periods. It is also vital to determine the impact of Cystic Fibrosis Transmembrane Conductance Regulator (CFTR) modulator therapy on lung inflammation in children. This novel class of treatments are the first to address the underlying defect in CF and have resulted in dramatic improvements when measured by outcomes such as lung function and pulmonary exacerbation rate.^5^ However, their impact on inflammation is less clear, with data suggesting they alter but may not fully resolve pulmonary inflammation.^6^ For children unable to access CFTR modulator therapy (either due to genotype or medication availability), anti-inflammatory therapies that ameliorate lung disease severity are desperately needed.

To date, there have been several barriers to investigating lung inflammation in young children with CF. This includes limited access to tissue-specific biospecimens, and where lung samples have been studied, they are often limited by small numbers of participants and an absence of controls. Further, historical analyses have largely been limited to the assessment of a small number of candidate cell types or soluble mediators. Lastly, due to the limited ability to perform lung function assessment in early life, understanding of the relationship between lung disease and inflammation in early life is challenging.

In this study, we combined paediatric respiratory and blood sampling with comprehensive clinical data and next-generation immunological techniques to extensively profile pulmonary and systemic inflammation in 70 preschool children with CF and a total of 32 age-matched preschool controls.

## Materials and methods

### Study participants

This study took place at the Royal Children’s Hospital (RCH, Melbourne, Australia) and involved analysis of bronchoalveolar lavage fluid (BAL) and blood samples from children with CF and controls. Table 1 describes the demographics and clinical characteristics of the study cohort. Samples were collected between 2017 and 2023. The CF group (total n=70) consisted of children who had surveillance bronchoscopy as part of clinical care through the AREST-CF programme,^2^ and the control group (total n=32) were children without CF who underwent general anaesthesia for the assessment and/or management of upper airway disease (such as stridor or tonsillar hypertrophy). To further determine the suitability of children in the control group, parents completed a questionnaire to assess for lung pathology and any child with a history of lung disease was excluded. Children had bronchoscopy performed at a time of clinical stability with no clinical features of acute infection or pulmonary exacerbation (in the case of children with CF) as judged by the clinical team.

**Table 1:** Demographics and clinical characteristics of the study cohort

	CF (no modulator) (CF.U)	CF.IVA	CF.LUM/IVA	Non-CF	P value*
BAL analytes					
Total number	54	11	5	23	
Sex: male, n (%)	23 (42.6)	8 (72.7)	2 (40)	18 (78.2)	0.012
Age: years, median (range)	2.0 (0.53–6.46)	4.0 (1.45–5.95)	4.5 (2.26–6.19)	3.47 (0.48–12.3)	0.013
Ethnicity: European, n (%)	47 (87)	11 (100)	5 (100)	19 (82.6)	0.631
CFTR mutation: Phe508 del-homozygote, n (%)	26 (48.1)	0 (0)	5 (100)		<0.0001
CFTR mutation: Phe509 del-heterozygote, n (%)	23 (42.6)	11 (100)	0 (0)		<0.0001
Diagnosis by newborn screening, n (%)	39 (72.2)	8 (72.2)	4 (80)		>0.999
Bronchiectasis at the time of sample, n (%)	16 (29.6)	1 (9.1)	1 (20)		0.485
Antibiotics (any) at the time of sample, n data available, n yes (%)	38, 22 (57.9)	9, 3 (33.3)	1, 0 (0)		0.201
Azithromycin at the time of sample, n data available, n yes (%)	38, 4 (10.52)	9, 0 (0)	1, 0 (0)		0.609
BAL flow cytometry					
Total number	30	2	6	8	
Sex: male, n (%)	17 (56.7)	2 (100)	3 (50)	5 (62.5)	0.824
Age: years, median (range)	2.0 (0.95–6.46)	5.16 (5.09–5.24)	4.74 (2.26–6.19)	3.03 (0.48–10.26)	0.03
Ethnicity: European, n (%)	24 (80)	2 (100)	6 (100)	8 (100)	0.492
CFTR mutation: Phe508 del-homozygote, n(%)	7 (23.3)	0 (0)	6 (100)		0.0006
CFTR mutation: Phe509 del-heterozygote, n(%)	19 (63.3)	2 (100)	0 (0)		0.002
Diagnosis by newborn screening, n (%)	21 (70)	2 (100)	5 (83.3)		0.814
Bronchiectasis at the time of sample, n (%)	7 (23.3)	0 (0)	2 (33.3)		0.784
Antibiotics (any) at the time of sample, n data available, n yes (%)	11, 3 (27.2)	1, 0 (0)	2, 0 (0)		>0.999
Azithromycin at the time of sample, n data available, n yes (%)	11, 1 (9.1)	1, 0 (0)	2, 0 (0)		>0.999
Plasma analytes					
Total number	24	3	4	32	
Sex: male, n (%)	13 (54.2)	2 (66.6)	2 (50)	20 (62.5)	0.874
Age: years, median (range)	1.98 (0.95–6.46)	4.96 (1.45–4.96)	4.26 (2.26–6.19)	3.4 (0.48–6.5)	0.06
Ethnicity: European, n (%)	21 (87.5)	3 (100)	4 (100)	25 (78.1)	0.733
CFTR mutation: Phe508 del-homozygote, n(%)	6 (25)	0 (0)	4 (100)		0.007
CFTR mutation: Phe509 del-heterozygote, n(%)	15 (62.5)	3 (100)	0 (0)		0.012
Diagnosis by newborn screening, n (%)	18 (75)	1 (33.3)	2 (50)		0.261
Bronchiectasis at the time of sample, n (%)	6 (25)	1 (33.3)	2 (50)		0.648
Antibiotics (any) at the time of sample, n data available, n yes (%)	10, 2 (20)	2, 2 (100)	2, 0 (0)		0.131
Azithromycin at the time of sample, n data available, n yes (%)	10, 1 (10)	2, 0 (0)	2, 0 (0)		>0.999

* Categorical variables were compared using Fisher’s exact tests and continuous variables were compared using Kruskal-Wallis tests.

BAL, bronchoalveolar lavage; CF, cystic fibrosis; CFTR, Cystic Fibrosis Transmembrane Conductance Regulator; IVA, ivacaftor; LUM, lumacaftor.

### Collection of samples and clinical information

Bronchoscopy and BAL were performed as previously described.^2^ In brief, three aliquots of sterile normal saline (1 mL/kg, max 20 mL per aliquot) were instilled into the right middle lobe, and another lavage of an aliquot was performed in the lingula or the site of greatest disease. This study used BAL from aliquots 2 and 3 from the right middle lobe. BAL was assessed for pathogens as per standard clinical testing in the RCH clinical microbiology laboratory, which included standard culture for bacteria and fungi as well as PCR testing for 11 viruses, *Bordetella pertussis* and *Mycoplasma pneumoniae*. Venous blood was also collected in EDTA tubes at the time of the procedure. Medication records were reviewed, and participants were classified as being treated or not treated with CFTR modulator therapy. At the time of the study, modulator therapies that were available to the participants were ivacaftor (IVA) (for children with an eligible class III or IV mutation), and lumacaftor (LUM)/IVA (for children homozygous for Phe508 del mutation). Children not on modulator therapy either had a genotype ineligible for modulator therapy or their parents had elected not to start treatment. To assess lung disease severity, each participant’s most recent CT scan was reviewed by a clinician trained in the assessment of early-life CF scans, and CF participants were classified as having bronchiectasis or not. Relevant metadata was also collected, including CFTR genotype (for CF participants) and parental report of participant ethnicity. For ethnicity data, the responses were mapped to the closest category in the Human Ancestry Ontology.^7^

### Processing of BAL and blood samples

BAL samples were immediately placed on ice and processed within 1 hour of the procedure. Blood samples were kept at room temperature and processed within 1 hour of the procedure. BAL samples were centrifuged at 300×*g* for 7 min at 4°C and the cell-free BAL supernatant collected and stored at −80°C. The cell pellet was resuspended in 10 mL media (RPMI supplemented with 2% fetal calf serum (FCS)), filtered through a 70 µm strainer, and centrifuged at 300×*g* for 7 min at 4°C. Cells were then aliquoted for flow cytometry. For blood samples, 100 µL of EDTA whole blood was aliquoted for flow cytometry. The remaining EDTA blood was centrifuged at 700×*g* for 10 min at room temperature and the supernatant (plasma) stored at −80°C. Our protocols for the collection and processing of paediatric BAL for single-cell analysis are publicly available at https://www.protocols.io/workspaces/earlyAIR.

### Flow cytometry of BAL and blood samples

Blood samples were lysed with 1 mL of red cell lysis buffer for 10 min at room temperature. Cells were washed with 1 mL phosphate-buffered saline (PBS) and centrifuged at 400×*g* for 5 min at room temperature. For both BAL and lysed whole blood cells, cells were resuspended in PBS for viability staining using near-infrared viability dye according to the manufacturer’s instructions. The viability dye reaction was stopped by the addition of FACS buffer (2% heat-inactivated FCS in PBS) and cells were centrifuged at 400×*g* for 5 min at 4°C. Cells were resuspended in 25 µL human FC-block (diluted 1:10) for 5 min at room temperature. The flow cytometry staining panel made up at 2× concentration was added 1:1 with the cells and incubated for 30 min on ice. Following staining, cells were washed with 1 mL FACS buffer and centrifuged at 400×*g* for 5 min at 4°C. Cells were resuspended in 150 µL FACS buffer for acquisition using a BD LSR X-20 Fortessa (online supplemental table 1) or a Cytek 5L Aurora (online supplemental table 2). The gating strategy used to define cell types obtained with these panels is depicted in online supplemental figures 1 and 2 (BAL and whole blood).

### Analyte quantification using Bio-Plex Pro panels

Cell-free BAL supernatant and plasma samples were thawed, and cytokines were measured in singlicate using the 48-plex Bio-Plex Pro Human Cytokine Screening panel (Bio-Rad, California, USA) and the 37-plex Bio-Plex Pro Human Inflammation panel (Bio-Rad, California, USA) according to the manufacturer’s instructions. BAL supernatants were run undiluted and plasma samples were diluted 1:5 in PBS. Immediately prior to the run, all samples were spun at 1000×*g* for 10 mins to remove particulates as per the recommendations. The following 78 cytokines were quantified: FGF basic, Eotaxin, G-CSF, GM-CSF, IFN-γ, IL-1β, IL-1ra, IL-1α, IL-2Rα, IL-3, IL-12 (p40), IL-16, IL-2, IL-4, IL-5, IL-6, IL-7, IL-8, IL-9, GRO-α, HGF, IFN-α2, LIF, MCP-3, IL-10, IL-12 (p70), IL-13, IL-15, IL-17A, IP-10, MCP-1 (MCAF), MIG, β-NGF, SCF, SCGF-β, SDF-1α, MIP-1α, MIP-1β, PDGF-BB, RANTES, TNF-α, VEGF, CTACK, MIF, TRAIL, IL-18, M-CSF, TNF-β, APRIL/TNFSF13, BAFF/TNFSF13B, sCD30/TNFRSF8, sCD163, Chitinase-3-like 1, gp130/sIL-6Rβ, IFN-β, sIL-6Rα, IL-11, IL-19, IL-20, IL-22, IL-26, IL-27 (p28), IL-28A/IFN-λ2, IL-29/IFN-λ1, IL-32, IL-34, IL-35, LIGHT/TNFSF14, MMP-1, MMP-2, MMP-3, Osteocalcin, Osteopontin, Pentraxin-3, sTNF-R1, sTNF-R2, TSLP and TWEAK/TNFSF12. Data were acquired on the Bio-Plex 200 system, and data QC was done using the Bio-Plex Manager 6.1 software (Bio-Rad). For data QC, standard curve outliers were removed, and individual standards were adjusted to achieve a recovery rate of 100±5% (observed concentration/expected concentration). A fit probability >0.9 was achieved for each standard curve. The concentration values of each analyte control were then compared with the expected concentration range specified on the assay data sheet for the control lot. Analytes that were not detected in >50% of samples were excluded from further analysis. For each analyte included in the analysis, samples that fell below the detection range were arbitrarily reported as half the lower limit of detection of the assay as previously described for this assay.^8^ After QC, 62 analytes remained in the analysis for BAL, and 52 analytes remained in the analysis for plasma. This final list of analytes is provided in online supplemental table 3.

### Data analysis

To identify relevant features in our high-dimensional data and focus our comparisons, principal components analysis (PCA) was conducted on analyte and cell data using the FactoMineR package in RStudio, including age, sex, bronchiectasis status and respiratory pathogen status as demographic variables. For respiratory pathogen status, we focused on four pathogens shown to be clinically important in early-life CF lung disease; these were *Pseudomonas aeruginosa*, *Staphylococcus aureus*, *Haemophilus influenzae* and *Aspergillus*.^9^ BAL samples were defined as either positive for one or more of the above important pathogens, positive for any virus (as determined by PCR) or as no pathogen detected, and these three variables were included in the PCA. Analyte levels (pg/mL) and cell populations (proportion of CD45+ live cells) in BAL and blood were compared between clinical groups using Mann-Whitney U tests. All p-values were corrected for false discovery rate using the Benjamini-Hochberg approach. An FDR-corrected p<0.1 and a fold change of >1.5 were required to reach statistical significance. Results are presented as volcano plots. Analytes that were significantly upregulated in the CF group underwent pathway analysis using the REACTOME database^10^ as well as protein-protein interactions (PPIs) analysis using STRING (V.12).^11^ Correlations between continuous variables were determined using two-tailed Spearman tests. Spearman p-values were corrected for false discovery rate using the Benjamini-Hochberg approach. An FDR-corrected p<0.1 was required to reach statistical significance. Hierarchical clustering analysis (complete clustering using Euclidean method) was performed on BAL analyte, BAL cell proportion and plasma analyte data from CF and control individuals and visualised in heatmaps. To visualise high-dimensional flow cytometry BAL data in two dimensions, the dimensionality reduction approach Uniform Manifold Approximation and Projection (UMAP) was applied to an equal number of cells per participant using default settings in the FlowJo V.10 UMAP plugin. Statistical analysis and data visualisation were performed in RStudio V.4.3.0, GraphPad Prism V.10.0.0, and FlowJo V.10.9.0.

## Results

### Soluble pulmonary immune signatures of early-life CF

**Figure 1:**
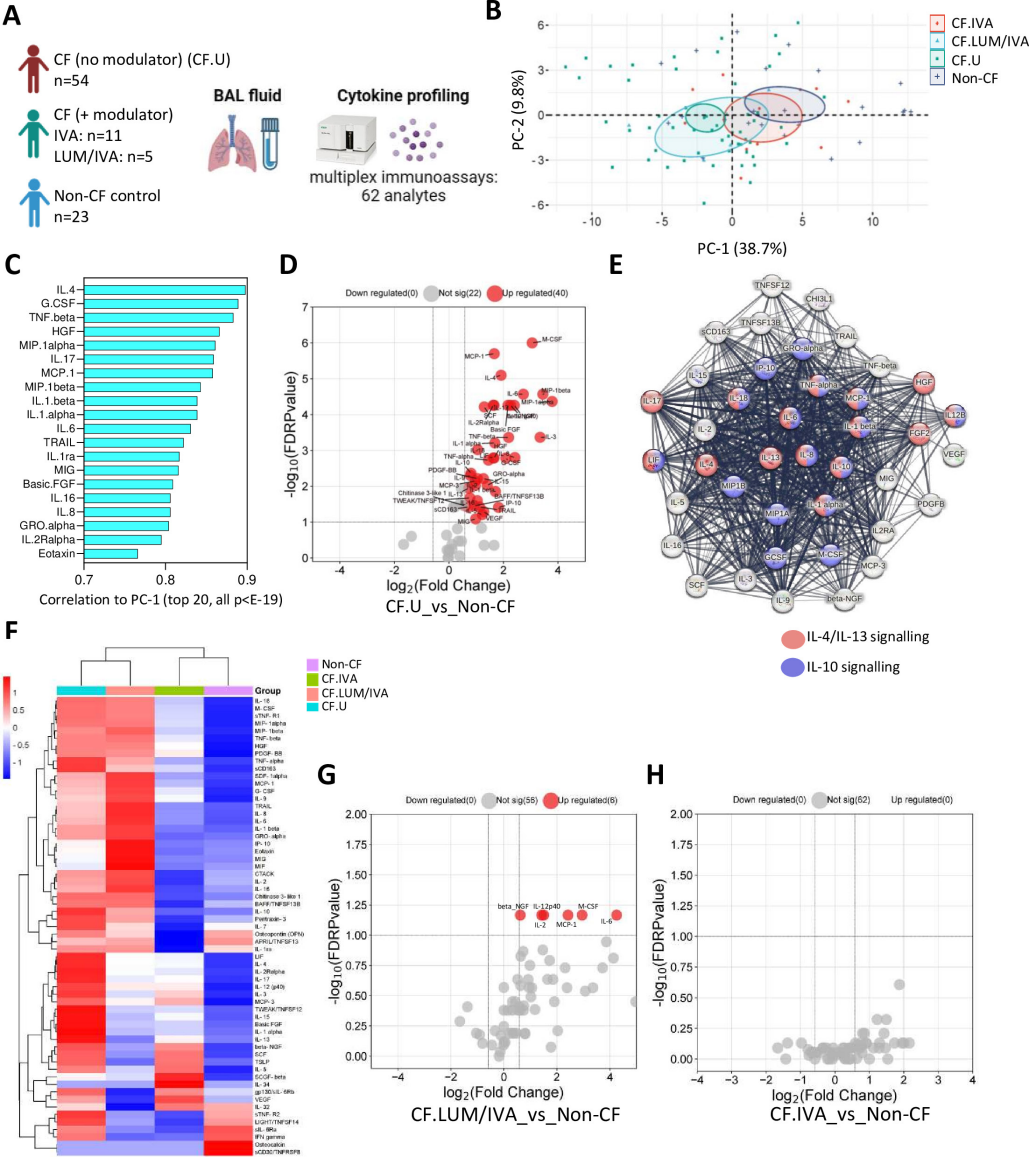
Soluble pulmonary immune signatures of early-life cystic fibrosis (CF). (**A**) Experimental workflow: bronchoalveolar lavage (BAL) cell-free fluid from children with CF (with (n=16) or without (n=54) Cystic Fibrosis Transmembrane Conductance Regulator (CFTR) modulator therapy) and non-CF controls (n=23) was analysed for immune-related analytes using commercially available multiplex immunoassays and the Bio-Plex 200 system. (**B**) Principal components analysis of BAL analyte data reveals clear separation between CF and non-CF samples, as well as between CF lumacaftor/ivacaftor (LUM/IVA) and CF IVA samples in principal component 1 (PC-1). (**C**) Correlation coefficients of the top 20 signatures that significantly correlated with PC-1 (all p<E-19). (**D**) Volcano plot depicting analytes that were significantly different in BAL cell-free fluid from children with CF (not on a modulator therapy) compared with controls. (**E**) Protein-protein interaction (PPI) analysis using STRING reveals significant interactions between analytes elevated in CF BAL (the strength of the line indicates the confidence of the interaction) and shows enrichment of interleukin (IL)-4/IL-13 and IL-10 signalling pathways in the data set. (**F**) Heatmap depicting unsupervised clustering analysis of median BAL cell-free fluid analytes in children with CF not on modulator therapy (blue), children with CF on LUM/IVA therapy (peach), children with CF on IVA therapy (green) and non-CF controls (purple). (**G**) Volcano plot depicting BAL cell-free fluid analytes that were significantly different in children with CF on LUM/IVA (n=5) therapy compared with non-CF controls (n=23). (**H**) Volcano plot depicting BAL cell-free fluid analytes that were significantly different in children with CF on IVA therapy (n=11) compared with non-CF controls (n=23) (none were significantly different). The p-values were calculated by Mann-Whitney U test and corrected for false discovery rate using the Benjamini-Hochberg approach. FDR-corrected p<0.1 and a fold change >1.5 were required to reach significance.

To extensively characterise the local immune environment in children with CF, we analysed 78 BAL immune-related analytes in 70 children with CF and 23 children with no history of lung disease (herein called non-CF controls) (figure 1A). Within the CF cohort, 54 children were not on any CFTR modulator therapy (CF no modulator), and 16 children were on a CFTR modulator therapy (CF+ modulator). The median age of children in the CF cohort was 2.3 years, and the median age of the control cohort was 3.4 years. Of the 78 analytes included in the multiplex assays, 62 were detected in BAL and included in downstream analysis (online supplemental table 3).

PCA was conducted on BAL analyte data from children with CF not on modulator therapy (denoted as CF.U, n=54); children with CF on CFTR modulator therapy LUM/IVA (denoted as CF.LUM/IVA, n=5) or IVA (denoted as CF.IVA, n=11) and age-matched controls (denoted non-CF, n=23) (figure 1B). This revealed separation between the CF and non-CF samples in the first dimension (PC-1), as well as between CF.LUM/IVA and CF.IVA samples, where CF.IVA were positioned more closely to the non-CF samples and CF.LUM/IVA were positioned more closely to the CF (no modulator) samples (figure 1B). A total of 55 analytes significantly correlated with PC-1, indicating widespread immune alteration in the CF group. The top 20 are shown in figure 1C (all p<E-19). The full list of signatures significantly correlated to PC-1 is provided in online supplemental table 4. Age, sex, bronchiectasis status or evidence of important respiratory pathogen (see ‘Materials and methods’) did not contribute significantly to this variation.

**Figure 2:**
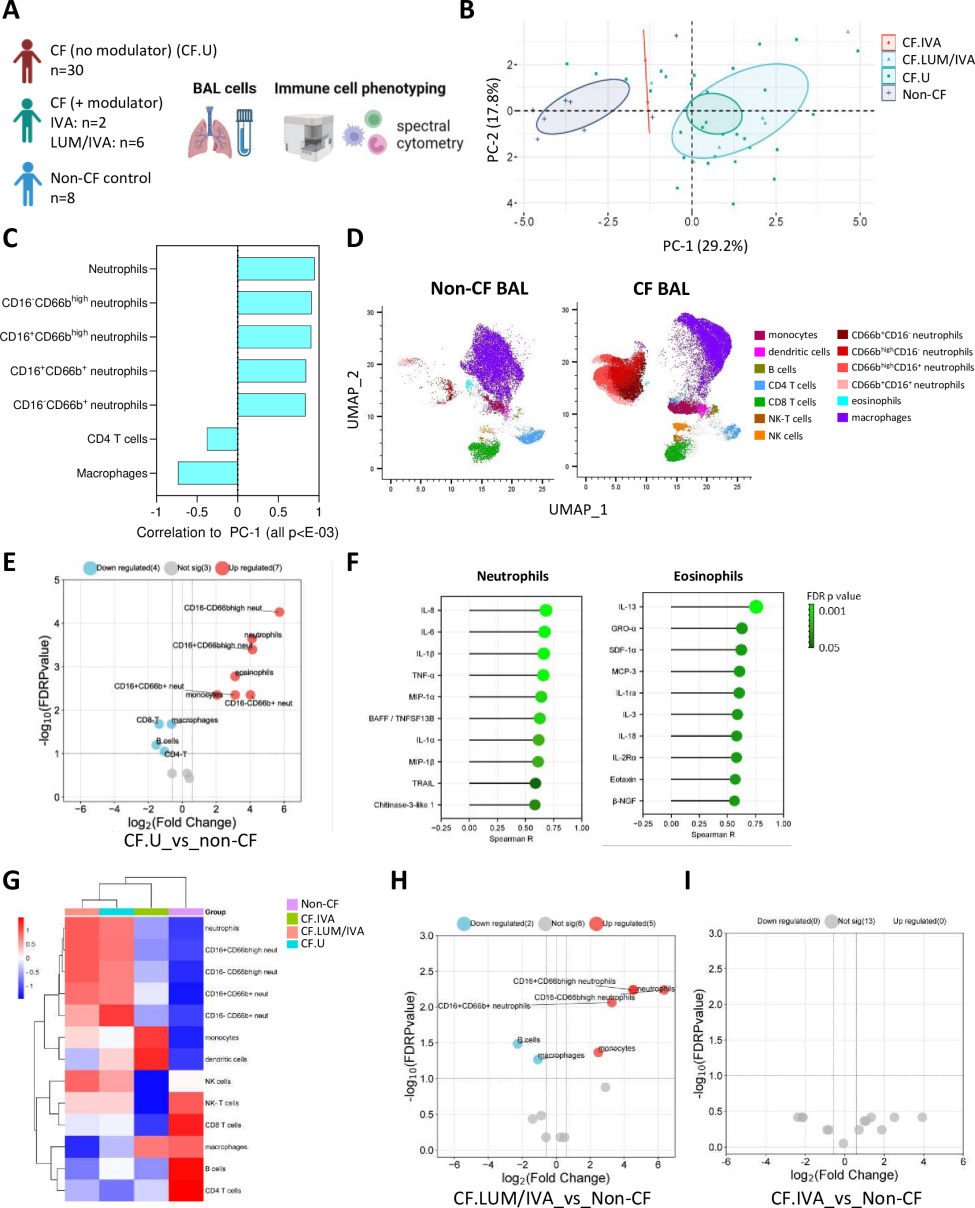
Cellular pulmonary signatures of early-life cystic fibrosis (CF). (**A**) Experimental workflow: bronchoalveolar lavage (BAL) cells from children with CF (with (n=8) or without (n=30) Cystic Fibrosis Transmembrane Conductance Regulator (CFTR) modulator therapy) and non-CF controls (n=8) were analysed by flow cytometry. (**B**) Principal components analysis of BAL cell data reveals clear separation between CF and non-CF samples, as well as between CF lumacaftor/ivacaftor (LUM/IVA) and CF IVA samples in principal component 1 (PC-1). (**C**) Correlation coefficients of the cellular signatures that significantly correlated with PC-1 (all p<E-13). (**D**) Uniform Manifold Approximation and Projection (UMAP) depicting the immune cell profile of BAL from children with CF (not on a CFTR modulator therapy) and non-CF controls. (**E**) Volcano plot depicting BAL immune cell populations that were significantly different in children with CF (not on a modulator therapy) compared with non-CF controls. (**F**) Two-tailed Spearman correlations of infiltrating granulocytes (neutrophils and eosinophils) and BAL cell-free fluid analytes. (**G**) Heatmap depicting unsupervised clustering analysis of median BAL immune cell populations in children with CF not on modulator therapy (blue), children with CF on LUM/IVA therapy (peach), children with CF on IVA therapy (green) and non-CF controls (purple). (**H**) Volcano plot depicting BAL immune cell populations that were significantly different in children with CF on LUM/IVA (n=6) therapy compared with non-CF controls (n=8). (**I**) Volcano plot depicting BAL immune cell populations that were significantly different in children with CF on IVA therapy (n=2) compared with non-CF controls (n=8) (none were significantly different). The p-values were calculated by Mann-Whitney U test and corrected for false discovery rate using the Benjamini-Hochberg approach. FDR-corrected p<0.1 and a fold change>1.5 were required to reach significance. Correlation p-values were determined by two-tailed Spearman test and corrected for false discovery rate using the Benjamini-Hochberg approach.

Confirming the PCA results, Mann-Whitney analysis of medians showed that 40 of these 55 analytes were significantly elevated in BAL of children with CF (no modulator) (CF.U) compared with age-matched controls (figure 1D). The full list of significant analytes is provided in online supplemental table 5. PPI analysis using STRING revealed that analytes elevated in the CF.U group were highly connected in significant interactive networks (PPI enrichment p-value<1.0e-16), and enrichment analysis of these networks using the REACTOME database revealed enrichment of both IL-4/IL-13 signalling pathway and the IL-10 signalling pathway (figure 1E).

To further explore the effect of CFTR modulator therapy on lung inflammation in preschool CF, we performed unsupervised hierarchical clustering analysis of median BAL analyte data from children with CF not on modulator therapy (CF.U) (n=54), children with CF on CFTR modulators LUM/IVA (LUM/IVA, n=5) or IVA (IVA, n=11) and controls (n=23). The two treated groups were similar in age (median 4.57 years for LUM/IVA and 4.1 years for IVA). Children in the LUM/IVA group had been on treatment for a median of 0.93 years while children in the IVA group had been on treatment for a median of 1.87 years. Those on IVA therapy clustered together with controls, while children on LUM/IVA therapy clustered together with CF children not on modulator therapy (figure 1F), mirroring what we observed earlier in the PCA. Participant-level clustering is shown in online supplemental figure 3A. We showed that six analytes remained significantly elevated in children on LUM/IVA therapy compared with controls (figure 1G). We showed no statistical difference in soluble lung immune profile between children on IVA therapy and controls (figure 1I). All data of figure 1 are provided in online supplemental appendix 1.

### Cellular pulmonary immune signatures of early-life CF

In addition to soluble analyte profiling of BAL cell-free fluid, we conducted flow cytometry analysis of freshly collected BAL cells from a subset of children in our cohort: 38 children with CF and 8 controls who had enough BAL collected for cellular analysis (figure 2A). Within the CF cohort, 30 children were not on any CFTR modulator therapy (CF.U), and 8 children were on a CFTR modulator therapy (CF+ modulator). The median age of children in the CF cohort was 2.6 years, and the median age of the control cohort was 3.0 years (figure 2A).

PCA was conducted on BAL flow cytometry data from children with CF not on modulator therapy (CF.U, n=30); children with CF on CFTR modulator therapy LUM/IVA (CF.LUM/IVA, n=6) or IVA (CF.IVA, n=2) and age-matched controls (non-CF, n=8) (figure 2B). As we observed with the BAL analyte data, this revealed separation between the CF and non-CF samples in the first dimension (PC-1), as well as between CF.LUM/IVA and CF.IVA samples (figure 2B). The cellular signatures that significantly correlated with PC-1 are shown in figure 2C, namely total neutrophil proportions and all neutrophil subsets in BAL which positively correlated with PC-1, and CD4 T cells and macrophages which negatively correlated with PC-1 (all p<E-03). Age, sex, bronchiectasis status or evidence of important respiratory pathogen did not contribute significantly to this variation.

Confirming the PCA results, Mann-Whitney analysis revealed that proportions of total neutrophils (median CF.U vs controls, 31.31 vs 1.81% of CD45+ cells, FDRp=0.0001) and all subsets of neutrophils (identified based on the expression of CD16 and CD66b activation markers) were elevated in CF.U compared with age-matched controls (figure 2D,E). Eosinophils (0.55 vs 0.06, FDRp=0.001) and monocytes (1.91 vs 0.45, FDRp=0.004) were also shown to be elevated in CF.U. Lower proportions of macrophages (51.62 vs 80.05, FDRp=0.02), CD8-T cells (1.49 vs 3.86, FDRp=0.01), B cells (0.33 vs 0.96, FDRp=0.06) and CD4-T cells (1.65 vs 3.41, FDRp=0.08) were also observed in CF.U compared with age-matched controls (figure 2E).

To understand the factors leading to infiltration of cells into the airway, we explored the relationships between innate immune cell frequency and soluble analyte concentration in BAL of children with CF (no modulator) (CF.U) (figure 2F). We found that neutrophilic infiltration in early-life CF was associated with elevated levels of 35 analytes in BAL. The top 10 most significant analytes are shown in figure 2F, and the complete list is provided in online supplemental table 6. We found that eosinophilic infiltration in early-life CF was associated with elevated levels of 19 analytes in BAL (figure 2F). The top 10 most significant analytes are shown in figure 2F, and the complete list is provided in online supplemental table 7.

Unsupervised hierarchical clustering analysis of median BAL immune cell population data also revealed that children on IVA therapy (n=2) clustered together with controls (n=8), while children on LUM/IVA therapy (n=6) clustered together with CF.U (n=30) (figure 2G). Participant-level clustering is shown in online supplemental figure 3B. We showed that total neutrophils, three subsets of neutrophils and monocytes remained elevated in children on LUM/IVA therapy compared with controls, and that macrophages and B cells were reduced (median CF LUM/IVA vs controls: neutrophils (42.7 vs 1.81, FDRp=0.005), monocytes (2.56 vs 0.45, FDRp=0.04), macrophages (37.79 vs 80.05, FDRp=0.05), B cells (0.19 vs 0.96, FDRp=0.03)) (figure 2H). We showed no difference in lung immune cells between children on IVA therapy and controls (figure 2I), although this analysis is limited by only n=2 children on IVA therapy with flow cytometry data. All data of figure 2 are provided in online supplemental appendix 1.

### Systemic immune signatures of early-life CF

**Figure 3:**
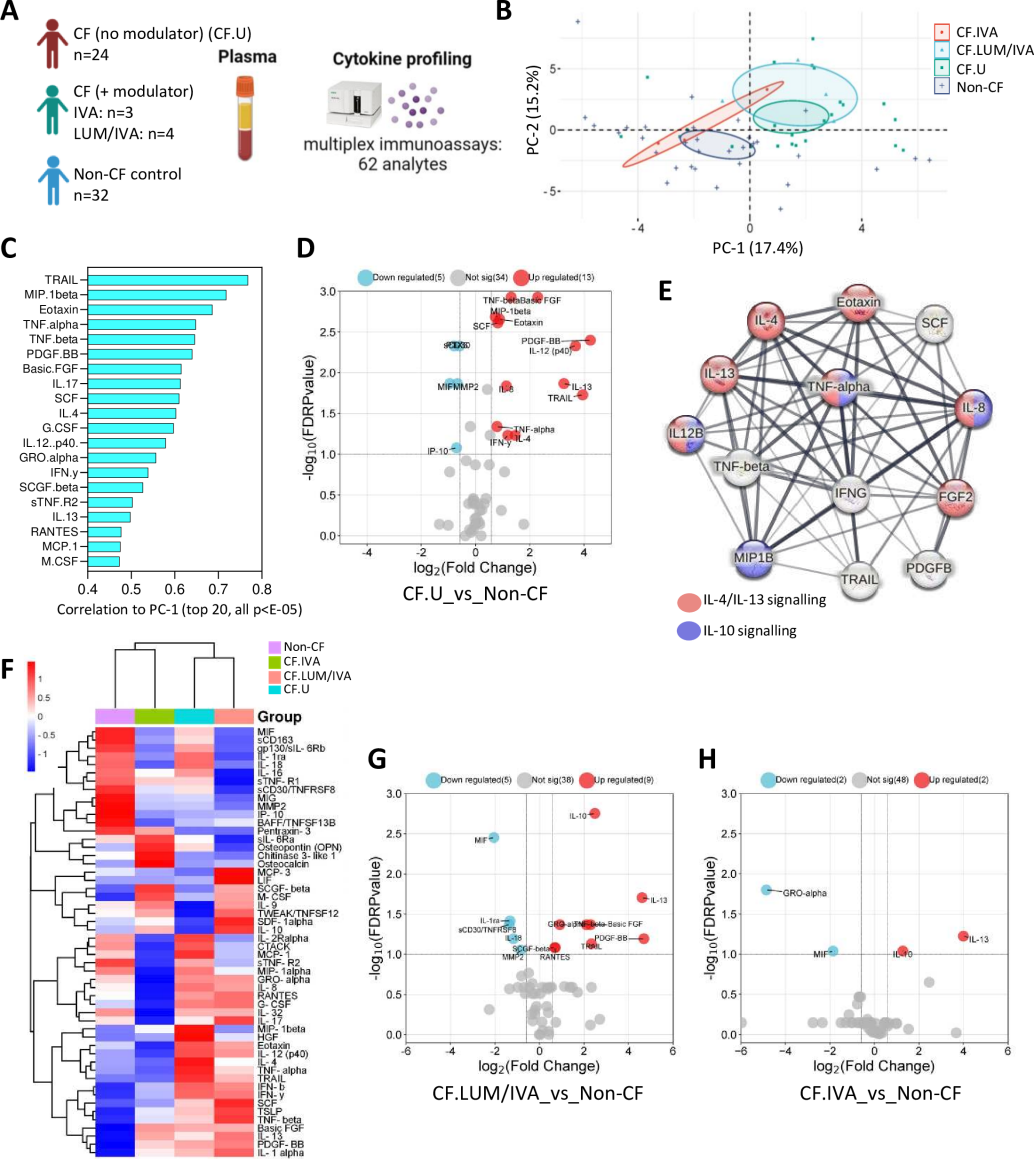
Systemic signatures of early-life cystic fibrosis (CF). (**A**) Experimental workflow: plasma from children with CF (with (n=7) or without (n=24) Cystic Fibrosis Transmembrane Conductance Regulator (CFTR) modulator therapy) and non-CF controls (n=32) was analysed for immune-related analytes using commercially available multiplex immunoassays and the Bio-Plex 200 system. (**B**) Principal components analysis of plasma analyte data reveals clear separation between CF and non-CF samples, as well as between CF lumacaftor/ivacaftor (LUM/IVA) and CF IVA samples in principal component 1 (PC-1). (**C**) Correlation coefficients of the top 20 signatures that significantly correlated with PC-1 (all p<E-05). (**D**) Volcano plot depicting analytes that were significantly different in plasma from children with CF (not on a modulator therapy) compared with controls. (**E**) Protein-protein interaction (PPI) analysis using STRING reveals significant interactions between analytes elevated in CF BAL (the strength of the line indicates the confidence of the interaction) and highlights interleukin (IL)-4/IL-13 and IL-10 signalling pathways as enriched in the data set. (**F**) Heatmap depicting unsupervised clustering analysis of plasma analytes in children with CF not on modulator therapy (blue), children with CF on LUM/IVA therapy (peach), children with CF on IVA therapy (green) and controls (purple). (**G**) Volcano plot depicting plasma analytes that were significantly different in children with CF on LUM/IVA therapy (n=4) compared with controls (n=32). (**H**) Volcano plot depicting plasma analytes that were significantly different in children with CF on IVA therapy (n=3) compared with controls (n=32). The p-values were calculated by Mann-Whitney U test and corrected for false discovery rate using the Benjamini-Hochberg approach. FDR-corrected p<0.1 and a fold change>1.5 were required to reach significance.

We next characterised systemic immunity in children with CF. We analysed 78 plasma immune-related analytes in 31 children with CF and 32 non-CF controls who had blood samples available for research (figure 3A). Within the CF cohort, 24 children were not on any CFTR modulator therapy (CF.U), and 7 children were on a CFTR modulator therapy (CF+ modulator). The median age of children in the CF cohort was 2.3 years, and the median age of the control cohort was 3.4 years. Of the 78 analytes included in the multiplex assays, 52 were detected in plasma and included in downstream analysis (online supplemental table 3).

PCA was conducted on plasma analyte data from children with CF not on modulator therapy (CF.U, n=24); children with CF on CFTR modulator therapy LUM/IVA (CF.LUM/IVA, n=4) or IVA (CF.IVA, n=3) and age-matched controls (non-CF, n=32) (figure 2B). As we observed with other data types in this cohort, this analysis revealed separation between the CF and non-CF samples in the first dimension (PC-1), as well as between CF.LUM/IVA and CF.IVA samples, where CF.IVA were positioned more closely to the non-CF samples and CF.LUM/IVA were positioned more closely to the CF.U samples (figure 2B). A total of 30 analytes significantly correlated with PC-1, and the top 20 are shown in figure 1C (all p<E-5). The full list of signatures significantly correlated to PC-1 is provided in online supplemental table 8.

Mann-Whitney analysis of medians showed that 13 of these 30 analytes were significantly elevated in the plasma of CF.U compared with age-matched controls, and 5 analytes were reduced (figure 3D). A full list of significant analytes is provided in online supplemental table 9. Of the 13 elevated plasma analytes, 11 were also shown to be significantly elevated in BAL of CF.U. This may suggest that plasma could act as a surrogate for lung inflammation in early-life CF. Further supporting this, PPI analysis using STRING revealed that plasma analytes elevated in the CF (no modulator) group were highly connected in significant interactive networks (PPI enrichment p-value<1.0e-16) and were enriched for both the IL-4/IL-13 and IL-10 signalling pathways (figure 3E), mirroring that observed for BAL analytes in figure 1.

Unsupervised hierarchical clustering analysis of median plasma analyte data revealed that children on IVA therapy clustered together with controls, while children on LUM/IVA therapy clustered together with CF.U (figure 3F). Participant-level clustering is shown in online supplemental figure 3C. We showed that nine analytes were significantly elevated in children on LUM/IVA therapy (n=4) compared with controls (n=32), and that five analytes were reduced (figure 3G). We showed that two analytes were elevated in children on IVA therapy (n=3) compared with controls (n=32); these were IL-13 and IL-10. GRO-α and MIF were reduced in children on IVA therapy compared with controls (figure 3H). All data of figure 3 are provided in online supplemental appendix 1.

### Colonisation with clinically important pathogens may not be a key contributor to early-life inflammation in the CF lung

Previous work has shown that four key pathogens (*P. aeruginosa*, *S. aureus*, *H. influenzae* and *Aspergillus*) uniquely contribute to infection burden and lung disease in young children with CF.^9^ Our multivariate analyses in figures1  2 showed, however, that the detection of one or more of these four key pathogens in BAL at the time of sampling was not a key contributor to the inflammatory response in early-life CF. This result prompted a further analysis into the interaction between the presence of pathogens in BAL in early life and inflammation in the CF lung.

**Figure 4:**
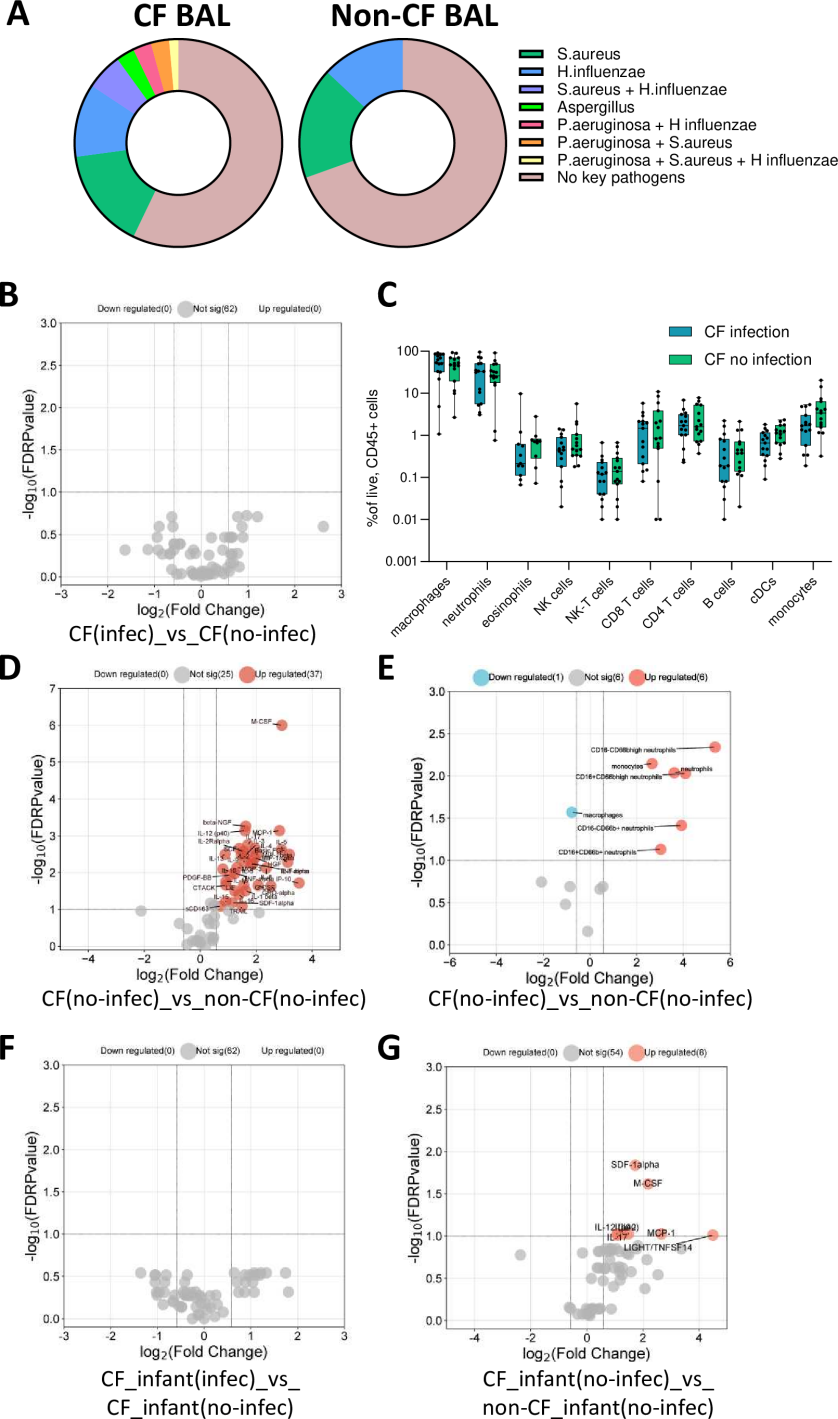
Relationship between clinically important pathogen colonisation and pulmonary inflammation in early-life cystic fibrosis (CF). (**A**) Proportion of colonisation with four key pathogens (*Pseudomonas aeruginosa*, *Staphylococcus aureus*, *Haemophilus influenzae* and *Aspergillus*) in CF and non-CF bronchoalveolar lavage (BAL) samples. (**B**) Volcano plot depicting BAL analytes that were significant between CF children who had evidence of one or more of these pathogens in BAL (n=22) and children with CF who had no evidence of these pathogens (n=32) (none were significant). (**C**) Box plots depicting immune cell populations in BAL of CF children who had evidence of one or more of these pathogens in BAL (n=15) and CF children who had no evidence of these pathogens in BAL (n=15). There were no significant differences. (**D**) Volcano plot depicting BAL analytes that were significant between CF children with no evidence of these pathogens (n=32) and non-CF children with no evidence of these pathogens (n=16). (**E**) Volcano plot depicting BAL immune cell populations that were significantly different between CF children with no evidence of these pathogens (n=15) and non-CF children with no evidence of these pathogens (n=7). (**F**) Volcano plot depicting BAL analytes that were significant between CF infants with evidence of these pathogens (n=12) and CF infants with no evidence of these pathogens (n=19) (none were significant). (**G**) Volcano plot depicting BAL analytes that were significant between CF infants with no evidence of these pathogens (n=19) and non-CF infants with no evidence of these pathogens (n=6). The p-values were calculated by Mann-Whitney U test and corrected for false discovery rate using the Benjamini-Hochberg approach. FDR-corrected p<0.1 and a fold change>1.5 were required to reach significance.

Clinical microbiology testing of CF BAL showed that 57.14% of participants had no evidence of the four key pathogens, 15.71% had *S. aureus* alone detected, 11.43% had *H. influenzae* alone detected, 2.85% had *Aspergillus* alone detected and a total of 12.85% had evidence of co-colonisation as shown in figure 4A. In the non-CF control BAL, 69.57% of participants showed no evidence of the four key pathogens, 17.39% had *S. aureus* alone detected and 13.04% had *H. influenzae* alone detected. There was no evidence of *Aspergillus* and no evidence of any co-colonisation of these four key pathogens in the non-CF control BAL (figure 4A). The number and type of pathogens detected in each CF and non-CF BAL sample are provided in online supplemental appendix 1.

Comparison of median BAL analyte levels between children with CF (no modulator) (CF.U) who had evidence of one or more of these pathogens in BAL (n=22) and CF.U who had no evidence of these pathogens in BAL (n=32) revealed no significant differences (figure 4B), suggesting a limited effect of these pathogens on the inflammatory profile in early-life CF. When analysing the BAL cellular data, the same result was observed, where there was no significant difference in BAL immune cell subset proportions between CF children who had evidence of these pathogens in BAL (n=15) and CF children who had no evidence of these pathogens in BAL (n=15) (figure 4C).

Next, we compared BAL analyte concentration in CF children with no evidence of these pathogens (n=32) to BAL analyte concentration in non-CF control children with no evidence of these pathogens (n=16) (figure 4D). This showed that children with CF without the four key pathogens present in their lungs still show a markedly elevated inflammatory response compared with non-CF control children, characterised by the elevation of 37 analytes in BAL. The BAL cellular data also showed similar results, where CF children with no evidence of these pathogens (n=15) showed marked infiltration of all inflammatory cell types compared with non-CF control children with no evidence of these pathogens (n=7) (figure 4E).

These results highlighted a potential role for intrinsic immune dysregulation in preschool CF that is independent of respiratory pathogen colonisation. To further explore this hypothesis and determine whether this response was evident as early as the first years of life, we compared BAL analyte levels from our youngest CF children aged between 0.5 and 2 years, with or without evidence of one or more of these pathogens in BAL (n=12 and n=19, respectively). This showed no significant differences between the groups (figure 4F). Finally, we compared BAL analyte concentration in CF children aged between 0.5 and 2 years with no evidence of these pathogens (n=19) to BAL analyte concentration in non-CF control children aged between 0.5 and 2 years with no evidence of these pathogens (n=6). This showed that infants with CF without the four key pathogens present in their lungs still show evidence of lung inflammation, characterised by the elevation of M-CSF, SDF-1alpha, IL-2, IL-12p40, IL-9, LIGHT/TNFSF14, IL-17 and MCP-1 in BAL (figure 4G).

Combined, this exploratory analysis shows that, in the current era of CF care, colonisation with clinically important pathogens does not significantly elevate inflammation in the preschool CF lung, and that inflammation in the CF lung is evident during infancy even in the absence of pathogen colonisation.

## Discussion

This study advances our understanding of early-life inflammation in the current era of CF, highlighting that inflammatory cell infiltration combined with a strong enrichment for type 2 inflammatory soluble signatures is found in the lungs and peripheral blood in the early preschool years, even in the absence of clinically important respiratory pathogen colonisation in the first two years of life. Further, the CFTR modulator IVA largely ameliorates pulmonary inflammation in early life, resulting in a profile similar to controls.

Alongside inflammatory cell infiltration, which has been observed previously in other work,^12 13^ we found elevation of 40 analytes in BAL of preschool children with CF. This is the first study to perform such a comprehensive and simultaneous assessment of the soluble mediators of inflammation, with the majority of previous studies only measuring one or few analytes in the same sample. The analytes we found to be elevated in CF were associated with traditional inflammation as observed previously, including TNF-α,^14^ IL-1β,^14^ IL-8,^15^,^19^ IL-6,^14^,^1620^ MIP-1α,^15^ MIG^15^ and MCP-1^15 16 21^; however, we also showed strong enrichment for type 2 inflammatory responses, including elevation of IL-4, IL-13, Eotaxin and FGF- 2^10^ in both BAL and peripheral blood, highlighting that these type 2 cytokines also play a key role in the establishment of inflammation in early CF.

We observed a strong association between neutrophil infiltration and key inflammatory cytokines, including IL-8, IL-6, IL-1β and TNF-α. Despite it being long acknowledged that neutrophilic inflammation is associated with irreversible structural lung damage in children with CF,^2^ there are no specific treatments to target this process currently in use. There are medications, such as brensocatib, which target neutrophilic inflammation currently undergoing assessment in clinical trials in CF and non-CF bronchiectasis.^22 23^ Another approach would be to repurpose an antibody which targets a cytokine associated with neutrophilic inflammation in early-life CF. Anakinra is an inhibitor of IL-1β (and IL-1α) which is currently being evaluated in people with CF; however, only patients older than 12 years are eligible for the trial.^24^ The findings of this study would suggest that anakinra may have a role in early-life CF, especially given the medication has been used in this age group for other conditions.^25^ There are also TNF-α inhibitors which are widely used, with some case reports of benefit in people with CF.^26 27^

As highlighted above, we also showed that cytokines associated with IL-4/IL-13 signalling were highly elevated in children with CF, were strongly associated with eosinophil recruitment and enriched in both BAL and blood. Dupilumab, a dual IL-4 and IL-13 inhibitor, was originally developed for the treatment of severe asthma, but has been repurposed for use in chronic obstructive pulmonary disease,^28^ with preliminary reports of benefit in non-CF bronchiectasis.^29^ Our work also directly answers a recent call for the identification of key type 2 signatures as attractive targets to decrease inflammation and fibrosis occuring in pulmonary tissue of patients with CF.^30^

Our finding that the CFTR modulator IVA was associated with a pulmonary and systemic immune profile close to that of controls is important for informing anti-inflammatory therapy for children receiving highly effective modulator therapy moving forward. To date, there has been contrasting evidence if CFTR modulators have an anti-inflammatory effect,^31^ and only a handful of studies have investigated the immune modifying effects of CFTR modulators in children. We have previously shown in a smaller cohort that IVA treatment was associated with reduced levels of IL-6, IL-8 and IL-1β in BAL,^32^ a finding that has also been supported in another study investigating these inflammatory mediators in nasal lavages from children on IVA therapy.^33^ Lepissier *et al* quantified inflammatory markers in sputum and blood in adolescents with CF and found that levels of IL-8, IL-1β and neutrophil elastase (NE) were reduced 1 month after initiation of elexacaftor/tezacaftor/ivacaftor (ETI) therapy.^34^ Bardin *et al* investigated mucosal-associated invariant T (MAIT) cells from children with CF and age-matched healthy controls.^35^ They observed a significant depletion of circulating MAIT cells prior to ETI treatment, and a significant increase in MAIT cell frequency and function after ETI initiation to the levels similar to healthy control children. Another finding of note was that participants treated with LUM/IVA clustered together with participants with CF not treated with a modulator. This is most likely explained by the modest efficacy of LUM/IVA, when measured by such endpoints as reduction in sweat chloride and improvement in pulmonary function, compared with other modulators such as IVA alone.^36^

The results of our study suggest that commencement of a highly effective modulator in early life, such as IVA or ETI, will likely result in an inflammatory environment similar to controls with some level of residual inflammation. Children on highly effective modulators will therefore likely need a different approach to children with CF who do not receive these treatments either due to ineligible genotype, intolerance or lack of access.

There is much debate about whether the proinflammatory state observed in people with CF is present as a result of infection^37 38^ or as a primary manifestation of abnormal CFTR function.^1439^,^42^ We found that colonisation with one or more of the four key pathogens shown to be clinically important in preschool CF (*P. aeruginosa*, *S. aureus*, *H. influenzae* and *Aspergillus*^9^) did not significantly influence cellular or soluble signatures of inflammation in BAL. We further showed that preschool children with CF without evidence of these pathogens show elevated pulmonary inflammation compared with non-CF controls, even if they are younger than 2 years old. This supports the alternative model of intrinsic dysregulation of inflammation that begins in early life. We acknowledge that CF children in our cohort without evidence of colonisation may have experienced prior infections that triggered the inflammatory response we observed; however, we performed a separate analysis on the 0.5–2-year-old group to try and address this point. We also recognise that we relied on results from clinical microbiology testing which is not as sensitive or specific as other methods.

Finally, we showed that inflammation extends beyond the lung and that a similar inflammatory signature of traditional and type 2 inflammation is present in the periphery of early-life CF. As the life expectancy of people with CF has improved, increased cardiovascular complications such as coronary artery disease and peripheral vascular disease have been observed.^43 44^ Early-life systemic inflammation is a known risk factor for the subsequent development of cardiovascular disease in later life.^45^ Our finding of systemic inflammation in the preschool period highlights that this process develops in early life for children with CF and strategies to target such inflammation, as well as protect cardiovascular health and other consequences of potential chronic systemic inflammation, need to be employed.

Our study has several strengths, including the use of tissue-specific samples, the inclusion of age-matched controls, the simultaneous assessment of over 60 soluble mediators and 13 immune cell populations, as well as an analytical approach that combined multivariate analysis, unsupervised testing and correction for multiple comparisons. The study also has limitations which primarily stem from the collection of BAL samples at the time of clinically indicated procedures with excess lavage fluid used for research purposes. This means that not every participant had all assessments performed, and there are different numbers of participants for each data type. Further, due to the constraints of the study, only BAL from the right middle lobe was available for analysis. There are data to suggest that the yield of BAL in terms of pathogen detection differs based on BAL location^46^; however, it was not possible in the current study to access BAL from multiple locations. Importantly, previous studies demonstrated increased structural lung disease in the right lung, compared with the left lung, so this study assessed the site of greatest disease.^46^ The cross-sectional design of the study is also a limitation as a longitudinal study with pre- and post-treatment samples is needed to definitively prove that IVA exerts anti-inflammatory effects. This study also assessed participants treated with modulators IVA and LUM/IVA. This was because, at the time of the study, these were the only two modulators available in Australia in the preschool age group. ETI has since become available to all children with at least one Phe508 del mutation, who are 2 years and older. IVA monotherapy is still used in children with eligible genotypes in the 4–23-month-old age group. While ETI has largely superseded both IVA and LUM/IVA, our findings offer insights into the association between therapies which target CFTR function and the impact on pulmonary and systemic inflammation. The fact that the control group is made up of children undergoing surgery for upper airway pathology is another limitation. To try and ensure the control group had no lung disease, we used a parent questionnaire and excluded any participants who did have report of lung pathology. Despite this, the control group may have undiagnosed lung disease, or alternatively their upper airway pathology may affect lower airway inflammation, both of which would result in them not representing true controls. This limitation reflects the difficulty in obtaining BAL samples from children for research.

In summary, our findings significantly advance the current understanding of both pulmonary and systemic inflammation in early-life CF. We highlight potential targets for anti-inflammatory therapy including the potential repurposing of existing therapies, as well as differences in inflammation between children on highly effective modulator therapies and those without. We also show elevated systemic inflammation in preschool children with CF, which may have long-term impacts on health. These results inform future research into the management of inflammation in CF and provide evidence for translating anti-inflammatory therapy into paediatric CF clinical care.

## Supplementary material

10.1136/thorax-2024-221634 online supplemental file 1

10.1136/thorax-2024-221634 online supplemental file 2

10.1136/thorax-2024-221634 online supplemental file 3

## Data Availability

All data relevant to the study are included in the article or uploaded as supplementary information.
