# Supplementary material for: Inflammation in preschool cystic fibrosis is of mixed phenotype, extends beyond the lung and is differentially modified by CFTR modulators
Source: Thorax. 2025 Feb 10;80(7):e221634. doi: 10.1136/thorax-2024-221634 (PMC12322466; doi:10.1136/thorax-2024-221634)
Supplement: online supplemental file 2 [file thorax-80-7-s002.pdf]

Supplementary Table 1. Flow cytometry panel for assessment of immune cell populations in BAL and blood (BD LSR Fortessa).

Antibody	Fluorophore	Volume in µL (2X in 100µL)	Supplier	Clone
CD16	BUV395	0.5	Becton Dickinson	3G8
CD56	BUV737	2	Becton Dickinson	NCAM16.2
CD11b	BUV805	2	Becton Dickinson	D12
CD206	BV421	1	Becton Dickinson	19.2
HLADR	V500	1	Becton Dickinson	G46-6
CD19	BV605	2	Becton Dickinson	SJ25C1
CD8	BV650	1	Becton Dickinson	RPA-T8
CD45	BV711	2	Becton Dickinson	HI30
CD14	BV786	4	Becton Dickinson	M5E2
CD3	BB515	2	Becton Dickinson	SK7
CD66b	PE	0.5	Becton Dickinson	G10F5
CD15	PE-CF594	1	Becton Dickinson	W6D3
CD11c	PE-Cy7	2	Becton Dickinson	B-ly6
CD63	A647	2	Becton Dickinson	H5C6
CD4	A700	2	Becton Dickinson	RPA-T4
Live/dead	NIR	N/A	Invitrogen (Thermo Fisher Scientific)	N/A

Supplementary Table 2. Flow cytometry panel for assessment of immune cell populations in BAL and blood (Cytek 5L Aurora).

Antibody	Fluorophore	Volume in µL (2X in 100µL)	Supplier	Clone
CD16	BUV395	0.5	Becton Dickinson	3G8
SIGLEC8	BUV661	1	Becton Dickinson	837535
CD56	BUV737	2	Becton Dickinson	NCAM16.2
CD11b	BUV805	2	Becton Dickinson	D12
CD206	BV421	1	Becton Dickinson	19.2
HLADR	V500	1	Becton Dickinson	G46-6
CD19	BV605	2	Becton Dickinson	SJ25C1
CD8	BV650	1	Becton Dickinson	RPA-T8
CD45	BV711	2	Becton Dickinson	HI30
CD66c	BV750	1	Becton Dickinson	B6.2/CD66
CD14	BV786	4	Becton Dickinson	M5E2
EPCAM	BB515	1	Becton Dickinson	EBA-1
CD3	FITC	2	Becton Dickinson	SK7
TSPAN8	BB700	2	Becton Dickinson	458811
CD66b	PE	0.5	Becton Dickinson	G10F5
CD15	PE-CF594	1	Becton Dickinson	W6D3
CD11c	PE-Cy7	2	Becton Dickinson	B-ly6
CD271	A647	2	Becton Dickinson	C40-1457
CD4	A700	2	Becton Dickinson	RPA-T4
Live/Dead	NIR	N/A	Invitrogen (Thermo Fisher Scientific)	N/A

Supplementary Table 3. Analytes included in the analysis after data processing and QC for BAL and plasma.

Analyte	BAL	Plasma	Analyte	BAL	Plasma
CTACK			TNF-beta		
Eotaxin			TRAIL		
Basic FGF			BAFF/TNFSF13B		
G-CSF			sCD30/TNFRSF8		
GRO-alpha			sCD163		
HGF			Chitinase 3-like 1		
IFN gamma			gp130/sIL-6Rb		
IL-1 alpha			sIL-6Ra		
IL-1ra			IL-32		
IL-2Ralpha			Osteocalcin		
IL-4			Osteopontin (OPN)		
IL-8			Pentraxin-3		
IL-9			sTNF-R1		
IL-10			sTNF-R2		
IL-12 (p40)			TSLP		
IL-13			TWEAK/TNFSF12		
IL-16			RANTES		
IL-17			IFN-beta		
IL-18			MMP2		
IP-10			IL-1 beta		
LIF			IL-2		
MCP-1			IL-3		
MCP-3			IL-5		
M-CSF			IL-6		
MIF			IL-7		
MIG			IL-15		
MIP-1alpha			beta-NGF		
MIP-1beta			VEGF		
PDGF-BB			APRIL/TNFSF13		
SCF			IL-34		
SCGF-beta			LIGHT/TNFSF14		
SDF-1alpha			MMP3		
TNF-alpha					

Supplementary Table 4. List of BAL analytes significantly correlated to PC-1

**BAL analyte**	**Correlation to PC-1**	**p.value**
IL.4	8.99E-01	2.53E-34
G.CSF	8.89E-01	1.42E-32
TNF.beta	8.84E-01	8.44E-32
HGF	8.65E-01	6.29E-29
MIP.1alpha	8.61E-01	2.04E-28
IL.17	8.59E-01	3.97E-28
MCP.1	8.58E-01	4.78E-28
MIP.1beta	8.43E-01	2.87E-26
IL.1.beta	8.41E-01	4.88E-26
IL.1.alpha	8.39E-01	8.49E-26
IL.6	8.31E-01	6.95E-25
TRAIL	8.22E-01	5.19E-24
MIG	8.17E-01	1.70E-23
IL.1ra	8.15E-01	2.78E-23
Basic.FGF	8.10E-01	8.01E-23
IL.8	8.08E-01	1.24E-22
IL.16	8.05E-01	2.37E-22
GRO.alpha	8.03E-01	3.56E-22
IL.2Ralpha	7.94E-01	2.29E-21
Eotaxin	7.64E-01	5.55E-19
TNF.alpha	7.57E-01	1.64E-18
BAFF.TNFSF13B	7.57E-01	1.68E-18
IL.9	7.55E-01	2.39E-18
SCF	7.52E-01	3.98E-18
IL.12..p40.	7.49E-01	6.28E-18
LIF	7.41E-01	2.13E-17
IP.10	7.28E-01	1.41E-16
Pentraxin.3	7.23E-01	2.87E-16
CTACK	7.22E-01	3.34E-16
IL.10	7.03E-01	3.77E-15
M.CSF	7.02E-01	4.33E-15
IL.18	6.62E-01	5.11E-13
sTNF.R1	6.53E-01	1.27E-12
IFN.gamma	6.23E-01	2.69E-11
IL.2	5.91E-01	4.31E-10
sIL.6Ra	5.80E-01	1.15E-09
sCD163	5.77E-01	1.47E-09
IL.3	5.65E-01	3.70E-09
Chitinase.3.like.1	5.56E-01	7.31E-09
sTNF.R2	5.47E-01	1.37E-08
SDF.1alpha	5.41E-01	2.14E-08
MCP.3	5.14E-01	1.35E-07

MMP3	5.13E-01	1.44E-07
PDGF.BB	4.78E-01	1.26E-06
MIF	4.77E-01	1.33E-06
IL.13	4.45E-01	8.05E-06
VEGF	4.02E-01	6.43E-05
sCD30.TNFRSF8	3.98E-01	7.90E-05
Osteopontin..OPN.	3.94E-01	9.29E-05
TSLP	3.80E-01	1.72E-04
TWEAK.TNFSF12	3.59E-01	4.16E-04
APRIL.TNFSF13	3.41E-01	8.36E-04
beta.NGF	3.40E-01	8.55E-04
IL.15	3.31E-01	1.18E-03
gp130.sIL.6Rb	2.76E-01	7.37E-03

Supplementary Table 5. List of significant analytes for BAL CF (no modulator) vs non-CF comparison (data associated with Figure 1B).

Significant analyte	CF (no modulator) [Median, pg/mL]	Non-CF [Median, pg/mL]	FDR P value
Basic FGF	16.41	3.655	0.000055
G-CSF	602.65	141.825	0.001622
GRO-alpha	1526.5	575.46	0.009345
HGF	57.565	18.265	0.001622
IL-1 alpha	22.95	7.14	0.000618
IL-1 beta	6.04	1.855	0.013946
IL-2	2.58	1.315	0.009456
IL-2Ralpha	9.04	2.93	0.000055
IL-3	0.66	0.065	0.00043
IL-4	2.07	0.55	0.000008
IL-5	104.29	44.7	0.061496
IL-6	18.455	2.785	0.000027
IL-8	348.795	65.325	0.001577
IL-9	8.505	4.925	0.005018
IL-10	3.69	2.105	0.004335
IL-12 (p40)	40.675	7.72	0.000055
IL-13	1.125	0.68	0.011305
IL-15	212.02	88.335	0.006115
IL-16	94.445	45.9	0.025141
IL-17	8.25	2.62	0.000055
IL-18	11.67	5.635	0.000948
IP-10	154.905	43.995	0.038732
LIF	26.37	8.55	0.001622
MCP-1	30.995	9.795	0.000002
MCP-3	2.16	1.11	0.00713
M-CSF	25.435	3.065	<0.000001
MIG	83.895	42.565	0.081599
MIP-1alpha	9.575	0.695	0.000043
MIP-1beta	60.11	5.525	0.000027
beta-NGF	3.115	0.65	0.000055
PDGF-BB	27.735	13.45	0.006115
SCF	27.355	11.145	0.000061
TNF-alpha	28.72	10.52	0.001868
TNF-beta	7.3	1.6	0.000435
TRAIL	39.155	17.96	0.037705
VEGF	74.885	30.92	0.050943
BAFF/TNFSF13B	1558.535	814.4	0.035085
sCD163	13392.15	8159.79	0.037705
Chitinase 3-like 1	5993.15	3975.36	0.035085
TWEAK/TNFSF12	37.165	21.79	0.021111

Supplementary Table 6. List of analytes significantly associated with neutrophilic infiltration in CF (data associated with Figure 2F).

Significant analyte	Spearman r	FDR P value
IL-8	0.681992	0.002198
IL-6	0.668856	0.002198
IL-1β	0.661741	0.002198
TNF-α	0.657909	0.002198
BAFF / TNFSF13B	0.626696	0.002541
MIP-1α	0.639847	0.002541
IL-1α	0.617216	0.00414
MIP-1β	0.611742	0.004201
Chitinase-3-like 1	0.583537	0.004906
TRAIL	0.588944	0.006055
HGF	0.57616	0.007516
G-CSF	0.570334	0.007907
M-CSF	0.546251	0.009781
MCP-1	0.546251	0.009781
FGF basic	0.54319	0.009781
IL-17	0.542716	0.009781
MMP-3	0.524642	0.009781
sTNF-R1	0.523915	0.009781
IL-4	0.538662	0.009781
TNF-β	0.537882	0.009781
MIG	0.5052	0.018022
LIF	0.480219	0.027337
SCGF-β	0.466338	0.033347
IL-12 (p40)	0.455703	0.03627
SDF-1α	0.454209	0.03627
VEGF	0.453749	0.03627
SCF	0.449555	0.03627
IL-18	0.445539	0.03627
Eotaxin	0.445387	0.03627
PDGF-BB	0.445358	0.03627
IL-2	0.442802	0.036575
IL-32	0.425693	0.036829
MCP-3	0.437876	0.037167
IL-9	0.433069	0.038907
sTNF-R2	0.402181	0.048855

Supplementary Table 7. List of analytes significantly associated with eosinophilic infiltration in CF (data associated with Figure 2F).

Significant analyte	Spearman r	FDR P value
IL-13	0.756713	0.010965
GRO-α	0.629825	0.061234
SDF-1α	0.623958	0.061234
MCP-3	0.608772	0.061234
IL-1ra	0.605263	0.061234
IL-3	0.589474	0.061234
IL-18	0.582456	0.061234
IL-2Rα	0.580957	0.061234
Pentraxin-3	0.551948	0.061234
Eotaxin	0.570175	0.061234
β-NGF	0.563653	0.061234
IP-10	0.559649	0.061234
IL-7	0.559017	0.061234
IL-17	0.551846	0.063334
IL-2	0.542105	0.068187
IL-4	0.529825	0.074527
IL-16	0.526316	0.074527
G-CSF	0.522807	0.074527
PDGF-BB	0.512506	0.081111

Supplementary Table 8. List of plasma analytes significantly correlated to PC-1.

**Plasma analyte**	**Correlation to PC-1**	**p.value**
TRAIL	7.68E-01	2.08E-13
MIP.1beta	7.18E-01	3.66E-11
Eotaxin	6.86E-01	5.33E-10
TNF.alpha	6.48E-01	9.52E-09
TNF.beta	6.47E-01	1.05E-08
PDGF.BB	6.40E-01	1.64E-08
Basic.FGF	6.15E-01	8.00E-08
IL.17	6.13E-01	9.14E-08
SCF	6.11E-01	1.08E-07
IL.4	6.03E-01	1.73E-07
G.CSF	5.97E-01	2.40E-07
IL.12..p40.	5.80E-01	6.48E-07
GRO.alpha	5.56E-01	2.19E-06
IFN.y	5.39E-01	5.29E-06
SCGF.beta	5.27E-01	9.31E-06
sTNF.R2	5.03E-01	2.66E-05
IL.13	4.99E-01	3.22E-05
RANTES	4.77E-01	7.74E-05
MCP.1	4.75E-01	8.30E-05
M.CSF	4.73E-01	9.04E-05
HGF	4.42E-01	2.85E-04
CTACK	4.05E-01	9.97E-04
SDF.1alpha	4.04E-01	1.02E-03
IL.10	3.74E-01	2.51E-03
IL.8	3.68E-01	3.05E-03
MIP.1alpha	3.66E-01	3.18E-03
sTNF.R1	3.55E-01	4.27E-03
TSLP	3.48E-01	5.23E-03
IL.2Ralpha	3.46E-01	5.46E-03
Osteopontin.OPN.	3.41E-01	6.17E-03

Supplementary Table 9. List of significant analytes for plasma CF (no modulator) vs non-CF comparison (data associated with Figure 4B).

Significant analyte	CF (no modulator) [Median, pg/mL]	Non-CF [Median, pg/mL]	FDR P value
Eotaxin	31.34	16.94	0.002213
Basic FGF	28.15	5.75	0.00118
IL-4	3.75	1.36	0.058397
IL-8	4.56	2.08	0.014565
IL-12 (p40)	73.31	5.69	0.004689
IL-13	4.12	0.43	0.013597
IP-10	126.26	206.14	0.083231
MIF	447.37	866.5	0.013597
MIP-1beta	276.68	167.39	0.002062
PDGF-BB	260.4	13.9	0.003991
SCF	44.41	25.17	0.002449
TNF-alpha	63.15	36.55	0.045712
TNF-beta	567.79	229.01	0.00118
TRAIL	23.78	1.54	0.018714
sCD30/TNFRSF8	2032.44	3070.54	0.004689
IFN-y	37.1	16.28	0.058397
MMP2	9736.645	15503.74	0.013597
Pentraxin-3	6542.705	11305.33	0.004689

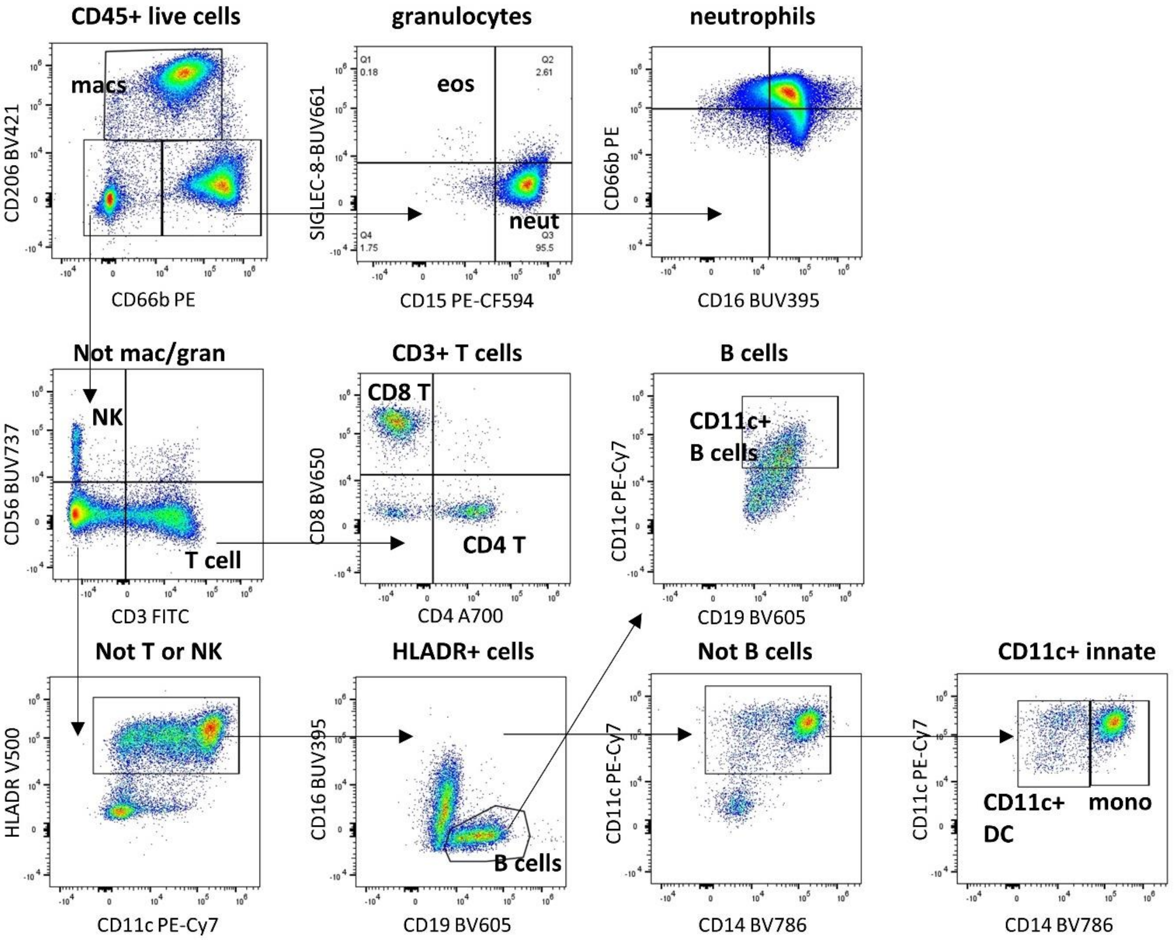

Supplementary Figure 1. Representative flow cytometry gating strategies for BAL. Within CD45+ live cells, macrophages were selected based on CD206 expression, and total granulocytes were selected based on CD66b expression. Within the granulocyte population, eosinophils were selected based on SIGLEC-8 expression and neutrophils were selected based on CD15 expression. Neutrophil subsets were identified using CD66b and CD16. CD206-CD66b- cells were subtyped into CD56+ NK cells, CD56+CD3+ NK-T cells, and CD3+ T cells. Within the CD3+ T cell fraction, CD4 and CD8 T cells were identified. HLADR+CD19+ cells were identified as B cells and subpopulation of B cells expressing CD11c+ were also gated. Monocytes were identified based on CD14 expression from within the CD11c+ HLADR+ population. Finally, CD11c+ DC were selected based on HLADR+CD14-CD11c+ expression pattern.

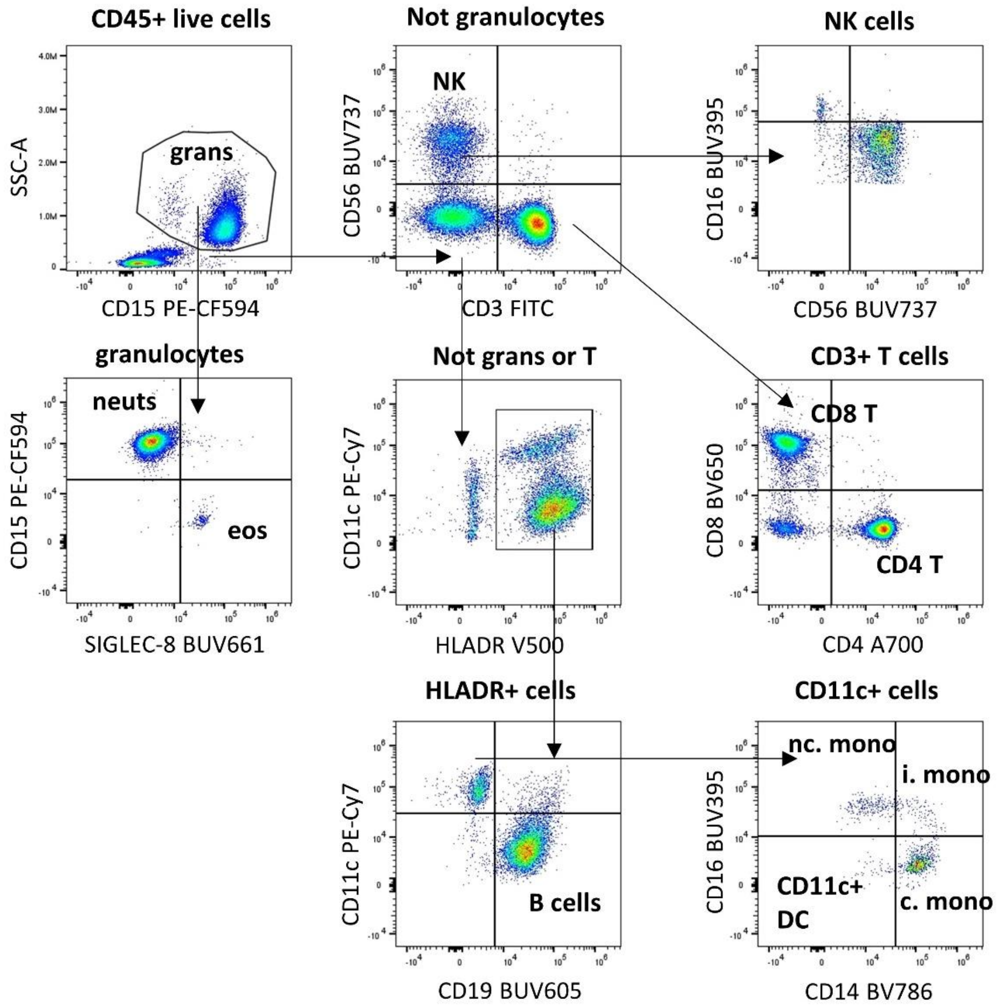

Supplementary Figure 2. Representative flow cytometry gating strategies for whole blood. Within CD45+ cells, granulocytes were selected based on a high SSC-A profile. Neutrophils were gated based on CD15+SIGLEC-8- expression, while eosinophils had low CD15 expression and were positive for SIGLEC-8. CD45+SSClow cells were further subtyped into CD56+ NK cells, CD56+CD3+ NK-T cells, and CD3+ T cells. Within the CD3+ T cell fraction, CD4 and CD8 T cells were identified. HLADR+CD19+ cells were identified as B cells and HLDR+CD11c+CD19- cells were identified as innate cells. Within the innate cell fraction, monocytes were selected based on CD14 expression while CD11c+ DCs were HLADR+CD11c+CD14-CD16- cells. Monocytes were assessed for CD16 expression, revealing classical (CD14+CD16-), intermediate (CD14+CD16+) and non-classical (CD14lowCD16+) populations.

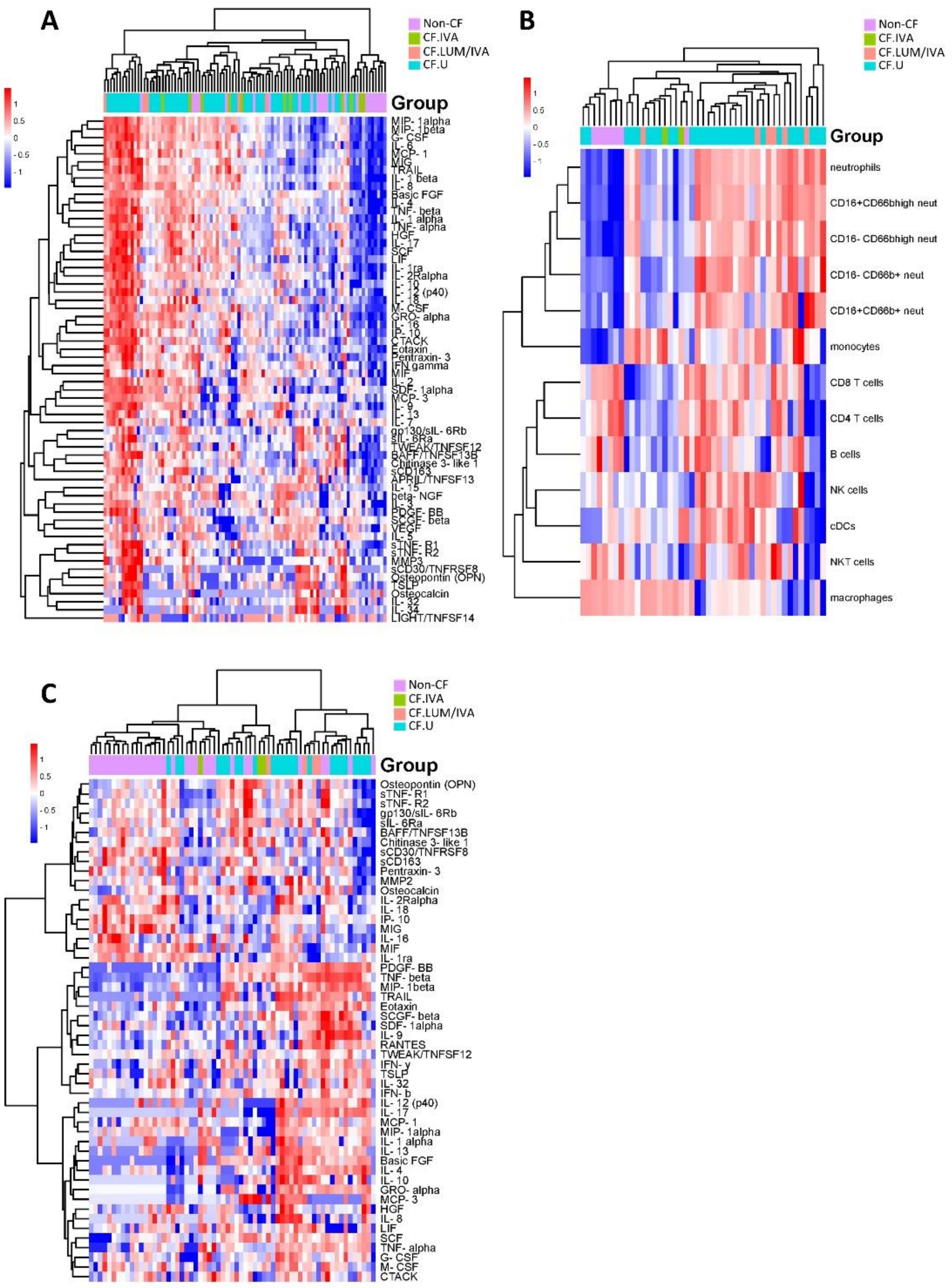

Supplementary Figure 3. Participant level clustering corresponding to heatmaps depicted in **(A)** Figure 1F (BAL analyte data), **(B)** Figure 2G (BAL cell data), and **(C)** Figure 3F (plasma analyte data).
