# Supplementary material for: Inflammation in preschool cystic fibrosis is of mixed phenotype, extends beyond the lung and is differentially modified by CFTR modulators
Source: Thorax. 2025 Feb 10;80(7):e221634. doi: 10.1136/thorax-2024-221634 (PMC12322466; doi:10.1136/thorax-2024-221634)
Supplement: online supplemental file 3 [file thorax-80-7-s003.pptx]

## Slide 1
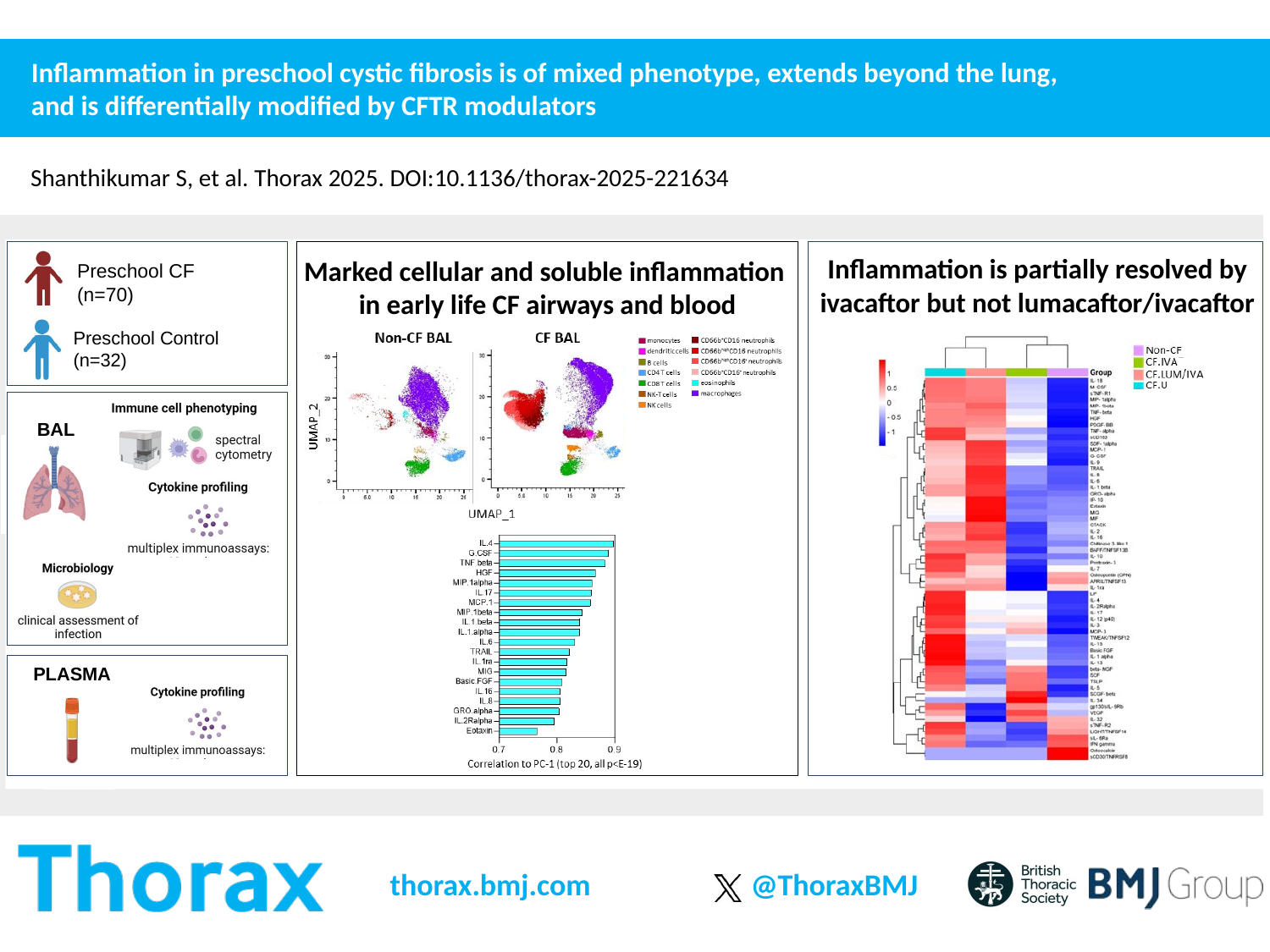

Inflammation in preschool cystic fibrosis is of mixed phenotype, extends beyond the lung,
and is differentially modified by CFTR modulators
Shanthikumar S, et al. Thorax 2025. DOI:10.1136/thorax-2025-221634
Inflammation is partially resolved by ivacaftor but not lumacaftor/ivacaftor
Marked cellular and soluble inflammation
in early life CF airways and blood
Preschool CF (n=70)
Manuscript Title
Preschool Control (n=32)
BAL
PLASMA
© Author(s) (or their employer(s) 2019. Re-use permitted under CC BY. Published by BMJ.
thorax.bmj.com @ThoraxBMJ
